# Design and Discovery of Quinazoline- and Thiourea-Containing Sorafenib Analogs as EGFR and VEGFR-2 Dual TK Inhibitors

**DOI:** 10.3390/molecules23010024

**Published:** 2017-12-23

**Authors:** Shaofeng Sun, Jingwen Zhang, Ningning Wang, Xiangkai Kong, Fenghua Fu, Hongbo Wang, Jianwen Yao

**Affiliations:** School of Pharmacy, Key Laboratory of Molecular Pharmacology and Drug Evaluation (Yantai University), Ministry of Education, Collaborative Innovation Center of Advanced Drug Delivery System and Biotech Drugs in Universities of Shandong, Yantai University, Yantai 264005, China; chemsunshaofeng@sina.com (S.S.); zhangjingwenydyy@163.com (J.Z.); wn573963424@163.com (N.W.); jwyao405@163.com (X.K);fenghua@luye.com (F.F.)

**Keywords:** quinazoline, thiourea, sorafenib, TK inhibitor, molecular docking

## Abstract

Both EGFR and VEGFR-2 play a critical role in tumor growth, angiogenesis and metastasis, and targeting EGFR and VEGFR-2 simultaneously represents a promising approach to cancer treatment. In this work, a series of novel quinazoline- and thiourea-containing sorafenib analogs (**10a**–**v**) were designed and synthesized as EGFR and VEGFR-2 dual TK inhibitors. Their in vitro enzymatic inhibitory activities against EGFR and VEGFR-2, and antiproliferative activities against HCT-116, MCF-7 and B16 cell lines were evaluated and described. Most of the compounds showed potent activities against both cell lines and TK kinases. Compounds **10b** and **10q** which exhibited the most potent inhibitory activities against EGFR (IC_50_ = 0.02 µM and 0.01 µM, respectively), VEGFR-2 (IC_50_ = 0.05 µM and 0.08 µM, respectively), and good antiproliferative activities, also displayed competitive anti-tumor activities than sorafenib in vivo by B16 melanoma xenograft model test.

## 1. Introduction

The epidermal growth factor receptor (EGFR) and vascular endothelial growth factor receptor (VEGFR-2), as potent targets for cancer therapy, have been proved to be crucial in signal transduction pathways involved in tumor cell proliferation, differentiation, migration and angiogenesis [1,2,3,4]. As a complicated signal network of interconnected circuits, EGFR and VEGFR-2 usually share common downstream signaling pathways. EGFR inhibition can decrease VEGF expression and attenuate angiogenesis, in the meantime leading to VEGFR-2 upregulation, and ultimately giving rise to the resistance of EGFR inhibitors [5,6]. Therefore, the combined inhibition of both EGFR and VEGFR-2 has thereby simultaneously become an efficient approach to cancer treatment with a synergistic effect [7,8].

**Figure 1:**
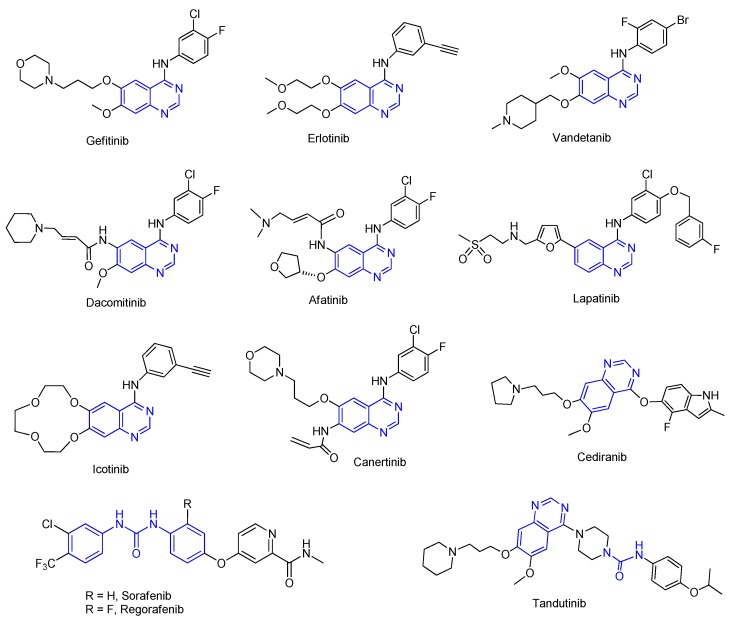
EGFR and/or VEGFR-2 tyrosine kinase inhibitors.

EGFR and VEGFR-2 share a catalytic domain that contains a cleft where adenosine triphosphate (ATP) binds. Based on this catalytic cleft, some small-molecule TK inhibitors have been developed and approved by the US Food and Drug Administration (FDA) in the past decade (Figure 1), which include gefitinib (AstraZeneca, London, UK, 2003), erlotinib (Genentech, South San Francisco, CA, USA and OSIP, Melville, NY, USA, 2004), sorafenib (Bayer, Leverkusen, Germany and Onyx, South San Francisco, CA, USA, 2005), vandetanib (Bristol-Myers Squibb, New York, NY, USA, 2006), lapatinib (GlaxoSmithkline, London, UK, 2007), regorafenib (Bayer, Leverkusen, 2012), and Afatinib (Boehringer Ingelheim, Ingelheim, Germany, 2013) [9,10,11,12,13]. Most of these tyrosine kinase inhibitors contain various quinazoline scaffolds that can interact with the ATP-binding pocket of EGFR and VEGFR-2.

**Figure 2:**
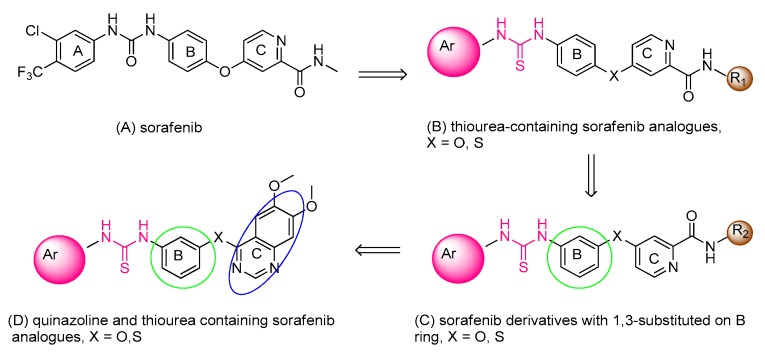
Modifications of sorafenib.

Sorafenib, a bisaryl ureas multikinase inhibitor, has been developed and launched for the treatment of renal cell carcinoma (RCC) and unresectable hepatocellular carcinoma (HCC). Due to its significant antitumor activities, more and more attention has been focused on the modification of sorafenib for years [14,15,16]. In our previous work [17,18,19,20], several series of diaryl-thiourea-containing sorafenib derivatives were designed and synthesized as antitumor agents (Figure 2); most compounds showed moderate to stronger in vitro antiproliferative activities, and some compounds exhibited potent inhibitory activities against the phosphorylation of VEGFR and the anti-angiogenic activities, which suggested that the thiourea moiety may improve the cytotoxicity of these compounds to some extent. Interestingly, compounds with 1,3-substitution on the B ring (Figure 2C) showed stronger anti-angiogenic activities than those with 1,4-substitution [18].

In an attempt to develop potent and selective antitumor agents, a series of quinazoline- and thiourea-containing sorafenib derivatives (as shown in Figure 2) were designed and synthesized, and their inhibitory activities against EGFR, VEGFR-2, and three cancer cell lines were evaluated. Moreover, the compounds **10b**, **10m** and **10q** were chosen to estimate their in vivo antitumor activity by a B16 melanoma xenograft model test. The compounds **10b** and **10q** exhibited the most potent inhibitory activities and better in vivo antitumor activities than sorafenib. Docking simulation was used to illustrate a common mode of interaction at the ATP binding site of EGFR and VEGFR-2.

## 2. Results and Discussion

### 2.1. Chemistry

**Scheme 1:**
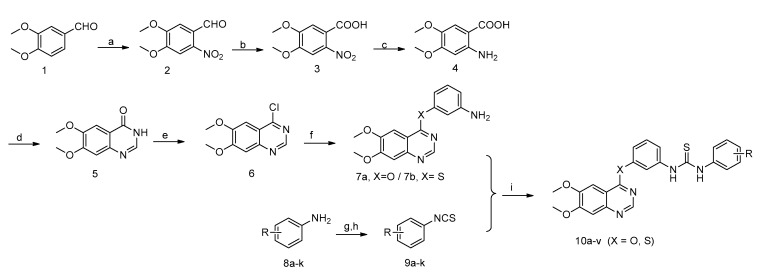
Reagents and conditions: (a) nitric acid (b) potassium permanganate, NaOH (c) H_2_; Pd/C (d) formamidine acetate (e) SOCl_2_; DMF (f) 3-amino phenol/3-amino thiophenol, KOBu-t, K_2_CO_3_; DMF (g) Dabco; CS_2_, toluene (h) BTC; CHCl_3_ (i) DCM.

The synthetic route to the target compounds was illustrated as outlined in Scheme 1.

2-Amino-4,5-dimethoxy-benzoic acid (Compound **4**) was synthesized from methylvanillin (Compound **1**) by nitration, oxidation and reduction, which was further treated with formamidine acetate in 140 °C to give 6,7-dimethoxy-3*H*-quinazolin-4-one (Compound **5**) in 95.7% yield. Compound **5** was reacted with SOCl_2_ to afford 4-chloro-6,7-dimethoxyquinazolin (Compound **6**) as a key intermediate of target compounds, which was condensed with 3-aminophenol or 3-aminothiophenol to give corresponding compounds **7a** and **7b** respectively. Furthermore, various substituted anilines (4-fluoro-3-trifluoromethylaniline, 4-chloro-3-trifluoromethylaniline, 4-bromo-3-trifluoromethylaniline, 3,5-di(trifluoromethyl)aniline, 3-trifluoromethylaniline, 4-trifluoromethoxyanoline, 2,4-dichloroaniline, 3,4-difluoroaniline, 4-chloroaniline, 4-fluoroaniline and 4-methylaniline) (**8a**–**k**) were reacted with CS_2_ and then treated with BTC to generate corresponding phenyl isothiocyanates (**9a**–**k**) in moderate to good yields. Finally, these phenyl isothiocyanates were reacted with each compound (**7a** or **7b**) in DCM at room temperature to afford target compounds (**10a**–**v**) in the total yields of 40.1% to 74.7%. The final products were purified by column chromatography and their structures were characterized by ^1^H-NMR and HRMS.

### 2.2. Biology

#### 2.2.1. EGFR and VEGFR-2 Inhibitory Assay

All the synthesized compounds (**10a**–**v**) were assayed with the enzymatic activities against EGFR and VEGFR-2 using sorafenib as a positive control. As shown in Table 1, most of tested compounds showed potent inhibitory activities against EGFR and VEGFR-2. Among them, compounds **10m** and **10q** displayed the most potent activity with IC_50_ of 0.01 μM against EGFR, and compound **10b** showed the most potent activity with IC_50_ of 0.05 μM against VEGFR-2, which is comparable to sorafenib with IC_50_ of 0.02 μM against EGFR and 0.08 μM against VEGFR-2 respectively.

Structure–activity relationships (SARs) were inferred from the data of the enzymatic experiment reported in Table 1. Compounds (**10a**, **10b**, **10c**, **10m**, **10o** and **10q**), containing two strong electron-withdrawing groups on the terminal aromatic ring, exhibited potent inhibitory activities against EGFR with IC_50_ values ranging from 0.01 to 0.05 μM, and against VEGFR-2 with IC_50_ values ranging from 0.05 to 0.19 μM. However, compounds **10f**, **10k**, **10n** and **10v** bearing electron-donating groups showed an obvious decrease of activities (IC_50_ more than 10 μM), The reasons may be as follows: (a) the compounds with electron-deficient terminal aromatic rings exist hydrophobic interaction with specific amino acid residues; (b) some electron-withdrawing groups (such as -F, -Cl, -Br, -CF_3_) on terminal aromatic rings can form hydrogen bonds by amino acid residues. Most of compounds **10l**–**v**, bearing diaryl thioether fragment, showed more potent activity against both EGFR and VEGFR-2 compared to the corresponding diaryl ether compounds **10a**–**k**, which suggested that the thioether moiety may improve the enzymatic inhibitory activity of these compounds. Moreover, the introduction of chlorine substituent at ortho-position of the thiourea group displayed weaker activity against both EGFR and VEGFR-2 (compounds **10g** versus **10i**, **10r** versus **10t**).

#### 2.2.2. In Vitro Antiproliferative Activity Assay

In vitro cell cytotoxicities of all the new compounds were initially evaluated against HCT116, MCF-7 and B16 cell lines by MTT assay using sorafenib as a positive control. The results were also summarized in Table 1. Most of the target compounds exhibited potent antiproliferative activities against all three cell lines. Among the tested compounds, compounds **10b**, **10c**, **10e**, **10l**, **10m**, **10o** and **10q** showed comparable antiproliferative activities to that of sorafenib and selective inhibitory activities against different cell lines. Compounds **10b** and **10q**, with the most potent EGFR/VEGFR-2 inhibitory activities, also displayed better potent antiproliferative activities against HCT-116, MCF-7 and B16 cell lines than sorafenib. The antiproliferative activities of the compounds were influenced by substituents on the terminal aromatic ring: (1) Compounds **10b**, **10c**, **10e**, **10l**, **10m**, **10o** and **10q** with strong electron-withdrawing groups (such as -F, -Cl, -Br, -CF_3_) on terminal aromatic ring exhibited potent antiproliferative activities against all three cell lines; (2) Compounds **10f**, **10k**, **10n** and **10v** containing electron-donating group (such as -CH_3_, -OCF_3_) showed relative weaker activity against the cancer cell lines. It indicated that the electron-withdrawing group on the terminal aromatic ring is also essential for the antiproliferative activities, and the antiproliferative activity of the title compounds is related to their dual EGFR/VEGFR-2 inhibitory activities.

#### 2.2.3. In Vivo Antitumor Activity Assay

The C57BL/6J mice were employed to establish the xenograft model of B16 melanoma, and the compounds **10b**, **10m** and **10q** were chosen to test their in vivo antitumor activity using sorafenib as a positive control. As shown in Table 2, compounds **10b**, **10m**, **10q**, and sorafenib can cause tumor regression, the growth of B16 tumors were inhibited at 31.25%, 49.22%, 20.31% and 64.06% by administering orally with sorafenib, **10b**, **10m**, and **10q** at 90 mg/kg, respectively. Compounds **10b** and **10q** displayed better inhibitory activities against B16 melanoma than that of sorafenib. No obvious weight loss was observed in all treated groups.

### 2.3. Molecular Docking Studies

In order to better understand the interaction between the title compounds and kinases, molecular docking studies on the potent representative compound **10q** were performed using the Tripos Sybyl-x2.0 software (2.0, Tripos Inc., St. Louis, MO, USA).

**Figure 3:**
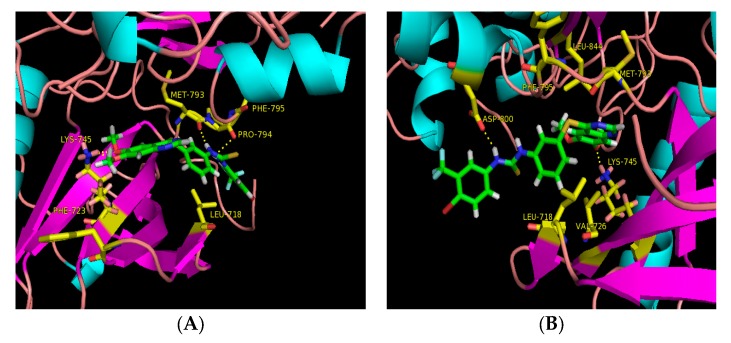
(**A**) 3D molecular docking model of compound **10q** with EGFR active site; (**B**) 3D model of the interaction between compound **10q** and VEGFR-2 ATP binding site. The hydrogen bonds are displayed as yellow dotted lines.

As shown in Figure 3, compound **10q** could be accommodated with EGFR comfortably (PDB: 2ity). the protonated N3 of quinazoline interacted in the EGFR ATP binding site with the amino acid Met-793 through an ionic bond. The NH of the thiourea were capable of forming hydrogen bonds with the amino acid residues Pro-794 and Met-793 respectively. Another hydrogen bond was formed between Lys-745 and the oxygen atoms of 6,7-position substituents of quinazoline. Hydrophobic interactions were observed with amino acid residues in the active site of EGFR, including Phe-795, Met-793 and Leu-718.

The binding model of compound **10q** into the ATP-binding cavity of VEGFR-2 kinase (PDB: 4asd) is also depicted in Figure 3. In this docking model, compound **10q** could be accommodated to the inactive DFG-out conformation of VEGFR-2 comfortably. The quinazoline and meta-disubstituted central phenyl were positioned in a hydrophobic pocket lined with Phe-795, Leu-844, Met-793, Val-726 and Leu-718. The hydrogen bond was formed between NH of the thiourea group and the amino acid residue Asp-800. In addition, 6,7-methoxy group of quinazoline could form another hydrogen bond with Lys-745.

## 3. Experimental Section

### 3.1. General Information

All the materials used were purchased from commercial suppliers and without further purification. Solvents were distilled before using and flash chromatography was performed using silica gel (60 Å, 200–300 mesh). All reactions were monitored by thinlayer chromatography on 0.25 mm silica gel plates (60GF-254) and visualized with UV light. Melting points were determined on an electrothermal melting point apparatus and were uncorrected. Proton nuclear magnetic resonance spectra were obtained on a Brucker Avance 400 spectrometer using TMS as an internal standard in DMSO-*d*_6_ or CDCl_3_ solutions. Chemical shifts were reported in delta (δ) units, parts per million (ppm) downfield from trimethylsilane. ESI-MS were determined on an API 4000 spectrometer. High-resolution mass spectral (HRMS) data were conducted by the Shandong Analysis and Test Center, and are reported as *m*/*z* (relative intensity).

#### 3.1.1. 4,5-Dimethoxy-2-nitrobenzaldehyde (**2**)

4,5-Dimethoxy benzaldehyde (16.6 g, 0.1 mol) was slowly added to 65% (g/g) nitric acid (100 mL) under stirring at a temperature range of 20–25 °C. The mixture was stirred for another 1 h and slowly added to 400 mL of ice water, a large amount of yellow solid was formed. The crude product was got by filtration and recrystallized in ethanol to get 4,5-Dimethoxy-2-nitrobenzaldehyde (Compound **2**) 16.0 g as a yellow solid, yield 78.2%, m.p. 133–135 °C.

#### 3.1.2. 4,5-Dimethoxy-2-nitrobenzoic acid (**3**)

Compound **2** (33.0 g, 156.3 mmol) was suspended in 0.6 M NaOH (500 mL) under stirring, KMnO_4_ (19.7 g, 125.0 mmol) was slowly added to the reaction mixture. After 7 h of stirring, the initial purple color went black, then a yellow solution was got after filtration. The pH value of the solution was adjusted to 2–3 by 10% HCl and large amounts of yellow solid was got. 4,5-Dimethoxy-2-nitro-benzoic acid (Compound **3**) was obtained after filtration as a yellow solid, yield: 83.0%, m.p. 193.2–195.0 °C.

#### 3.1.3. 2-Amino-4,5-dimethoxy-benzoic acid (**4**)

Compound **3** (10 g, 44.02 mmol), Pd/C (0.5 g) and 200 mL methanol were added to the 500 mL high pressure reactor, the air in the reactor was replaced by H_2_ (0.1 MPa) three times and shut down the vent valve. The mixture was stirred at 45 °C and 0.4 MPa of H_2_ for 2 h, after the reaction was over, Pd/C was filtered out and the solution was distilled under vacuum as soon as possible. The resulting residue was filtered and washed to give a yellow solid 7.2 g with a yield of 83.7%, m.p. 170.4–172.0 °C.

#### 3.1.4. 6,7-Dimethoxy-3*H*-quinazolin-4-one (**5**)

Compound **4** (5.0 g, 25.38 mmol) and formamidine acetate (4.0 g, 38.83 mmol) were dissolved in 5 mL of DMSO and heated to 140–170 °C under stirring. The initial black color went brown and some solid was formed. The reaction mixture was constantly stirred for 4h and then cooled to 25 °C. 50 mL H_2_O was added to the mixture and the product was obtained by filtration as a light yellow solid 5.0 g with a yield of 95.7%, m.p. 295.5–297.0 °C.

#### 3.1.5. 4-Chloro-6,7-dimethoxy-quinazoline (**6**)

A solution of compound **5** (1.7 g, 8.2 mmol) in SOCl_2_ (15 mL) containing 0.05 mL of *N,N*-dimethylformamide (DMF) were heated to reflux in 100 mL flask for 1 h. Excess SOCl_2_ was distilled in vacuum and the resulting residue was adjusted to pH value 8–10 with aqueous Na_2_CO_3_. 1.53 g of product was got after filtered as yellow solid, yield: 82.2%, m.p. 180.4–182.0 °C.

#### 3.1.6. 3-(6,7-Dimethoxyquinazolin-4-yloxy)-benzenamine (**7a**)

A solution of 3-aminophenol (0.35 g, 3.2 mmol) in dry DMF (20 mL) was treated with potassium tert-butoxide (0.44 g, 3.9 mmol), and the mixture was stirred at room temperature for 1 h under nitrogen atmosphere, then a solution of **6** (0.76 g, 3.4 mmol) in DMF (50 mL) and potassium carbonate were added respectively. The mixture was heated to 80–85 °C for 2 h. After the temperature was cooled to room temperature, the mixture was diluted with water (100 mL) and extracted with EtOAc (3 × 100 mL). The extract was washed with brine (2 × 100 mL), dried over anhydrous sodium sulfate, and concentrated under reduced pressure to give light-brown solid **7a** 0.74 g, yield: 77.9%, m.p. 183.5–185.0 °C. ^1^H-NMR (400 MHz, DMSO-*d*_6_) δ 3.97 (s, 3H, O-CH_3_), 3.98 (s, 3H, O-CH_3_), 5.28 (s, 2H, -NH_2_), 6.98 (dd, *J* = 1.6 Hz, 9.6 Hz, 1H, Ph-H-6), 7.13 (d, *J* = 7.6 Hz, 1H, Ph-H-4), 7.23 (d, *J* = 1.6 Hz, 1H, Ph-H-2), 7.43 (t, *J* = 7.8 Hz, 1H, Ph-H-5), 7.55 (s, 1H, quinazoline H-5), 7.63 (s, 1H, quinazoline H-8), 8.56 (s, 1H, quinazoline H-2). HRMS (AP-ESI) *m*/*z*: calcd. for C_16_H_15_N_3_O_3_ [M + H]^+^ 298.1192, found 298.1199.

#### 3.1.7. 3-(6,7-Dimethoxyquinazolin-4-ylsulfanyl)-benzenamine (**7b**)

The synthesis is analogous to **7a** with 3-aminethiophenol as the starting material. The product was obtained as light yellow solid, yield: 74.0%, m.p. 172.4–175.2 °C. ^1^H-NMR (400 MHz, DMSO-*d*_6_) δ 3.97 (s, 3H, O-CH_3_), 3.98 (s, 3H, O-CH_3_), 5.33 (s, 2H, -NH_2_), 6.68 (dd, *J* = 1.6 Hz, 9.6 Hz, 1H, Ph-H-6), 6.73 (d, *J* = 7.6 Hz, 1H, Ph-H-4), 6.80 (d, *J* = 1.6 Hz, 1H, Ph-H-2), 7.13 (t, *J* = 7.8 Hz, 1H, Ph-H-5), 7.31 (s, 1H, quinazoline H-5), 7.33 (s, 1H, quinazoline H-8), 8.70 (s, 1H, quinazoline H-2). HRMS (AP-ESI) *m*/*z*: calcd. for C_16_H_15_N_3_O_2_S [M + H]^+^ 314.0963, found 314.0958.

#### 3.1.8. Substituted Phenyl Isothiocyanates (**9a**–**k**)

The synthesis of compounds (**9a**–**k**) were the same as the method described in our previous article [20], the crude products were purified by chromatography (ethyl acetate/petroleum ether = 1:1) on silica gel to afford corresponding phenyl isothiocyanates in moderate to good yields.

#### 3.1.9. Quinazoline and thiourea-containing sorafenib analogs (**10a**–**v**)

*1-[3-(6,7-Dimethoxyquinazolin-4-yloxy)phenyl]-3-(4-fluoro-3-trifluoromethylphenyl)thiourea* (**10a**). Compound **7a** (0.50 g, 1.65 mmol) was dissolved in 10 mL of dichloromethane (DCM) and the solution was cooled to 0–5 °C in ice bath under stirring, a solution of **9a** (0.38 g, 1.76 mmol) in DCM were added dropwise. The reaction mixture was stirred at room temperature for 16 h and the precipitation appeared. The suspension was filtered and washed with DCM to give light grey solid **10a** 0.51 g, yield: 59.1%, m.p. 130.5–133.0 °C, purity: 98.0% (HPLC). ^1^H-NMR (400 MHz, DMSO-*d*_6_) δ 3.98 (s, 3H, quinazoline -OCH_3_-6), 3.99 (s, 3H, quinazoline -OCH_3_-6), 7.11 (d, *J* = 7.8 Hz, 1H, Ph-H-6), 7.39–7.51 (m, 5H), 7.55 (s, 1H, quinazoline H-8), 7.77–7.81 (m, 1H, Ph’-H-2), 7.95 (dd, *J* = 2.5, 6.4 Hz, 1H, Ph’-H-6), 8.56 (s, 1H, quinazoline H-2), 10.16 (s, 1H, -NHCS-), 10.28 (s, 1H, -NHCS-). HRMS(AP-ESI) *m*/*z*: calcd. for C_24_H_19_F_4_N_4_O_3_S [M + H]^+^ 519.1114, found 519.1121.

The other compounds of this series were synthesized following the general procedure as described above.

*1-(4-Chloro-3-trifluoromethylphenyl)-3-[3-(6,7-dimethoxyquinazolin-4-yloxy)phenyl]thiourea* (**10b**). Off white solid, yield: 65.5%, m.p. 140.1–141.0 °C, purity: 98.1% (HPLC). ^1^H-NMR (400 MHz, DMSO-*d*_6_) δ 3.96 (s, 3H, quinazoline -OCH_3_-6), 3.97 (s, 3H, quinazoline -OCH_3_-7), 7.11 (d, *J* = 7.8 Hz, 1H), 7.33–7.48 (m, 3H), 7.52 (d, *J* = 4.9 Hz, 2H), 7.65 (d, *J* = 8.7 Hz, 1H), 7.79 (dd, *J* = 2.6, 8.4 Hz, 1H), 8.07 (d, *J* = 2.6 Hz, 1H), 8.54 (s, 1H), 10.20 (s, 1H, -NHCS-), 10.27 (s, 1H, -NHCS-). HRMS(AP-ESI) *m*/*z*: calcd. for C_24_H_19_ClF_3_N_4_O_3_S [M + H]^+^ 535.1418, found 535.0925.

*1-(4-Bromo-3-trifluoromethylphenyl)-3-[3-(6,7-dimethoxyquinazolin-4-yloxy)phenyl]thiourea* (**10c**). Off white solid, yield: 65.6%, m.p. 186.3–188.0 °C, purity: 98.0% (HPLC). ^1^H-NMR (400 MHz, DMSO-*d*_6_) δ 3.96 (s, 3H, quinazoline -OCH_3_-6), 3.97 (s, 3H, quinazoline -OCH_3_-7), 7.11 (d, *J* = 7.8 Hz, 1H), 7.36 (s, 1H), 7.39–7.48 (m, 2H), 7.50–7.59 (m, 2H), 7.71 (dd, *J* = 8.5, 2.6 Hz, 1H), 7.81 (d, *J* = 8.5 Hz, 1H), 8.09 (d, *J* = 2.5 Hz, 1H), 8.55 (s, 1H), 10.29 (s, 1H, -NHCS-), 10.34 (s, 1H, -NHCS-). HRMS(AP-ESI) *m*/*z*: calcd. for C_24_H_19_BrF_3_N_4_O_3_S [M + H]^+^ 580.0313, found 580.0318.

*1-(3,5-Ditrifluoromethylphenyl)-3-[3-(6,7-dimethoxyquinazolin-4-yloxy)phenyl]thiourea* (**10d**). Off white solid, yield: 47.6%, m.p. 122.8–125.6 °C, purity: 98.4% (HPLC). ^1^H-NMR (400 MHz, DMSO-*d*_6_) δ 3.98 (s, 3H, quinazoline -OCH_3_-6), 3.99 (s, 3H, quinazoline -OCH_3_-7), 7.15 (d, *J* = 7.8 Hz, 1H, Ph-H-6), 7.40 (s, 1H), 7.41 (d, *J* = 7.8 Hz, 1H), 7.49 (t, *J* = 7.8 Hz, 1H), 7.53 (s, 1H), 7.55 (s, 1H), 7.81 (s, 1H), 8.26 (s, 2H), 8.56 (s, 1H), 10.42 (s, 1H, -NHCS-), 10.49 (s, 1H, -NHCS-). HRMS(AP-ESI) *m*/*z*: calcd. for C_25_H_19_F_6_N_4_O_3_S [M + H]^+^ 569.1082, found 569.1078.

*1-(3-Trifluoromethylphenyl)-3-[3-(6,7-dimethoxyquinazolin-4-yloxy)phenyl]thiourea* (**10e**). White solid, yield: 71.7%, m.p. 147.5–149.6 °C, purity: 98.7% (HPLC). ^1^H-NMR (400 MHz, DMSO-*d*_6_) δ 3.96 (s, 3H, quinazoline -OCH_3_-6), 3.97 (s, 3H, quinazoline -OCH_3_-7), 7.07–7.11 (m, 1H), 7.38 (d, *J* = 4.8 Hz, 2H), 7.42–7.47 (m, 2H), 7.52–7.58 (m, 3H), 7.74 (dd, *J* = 2.4, 7.4 Hz, 1H), 7.94 (s, 1H), 8.55 (s, 1H), 10.12 (s, 1H, -NHCS-), 10.17 (s, 1H, -NHCS-). HRMS(AP-ESI) *m*/*z*: calcd. for C_24_H_20_F_3_N_4_O_3_S [M + H]^+^ 501.1208, found 501.1210.

*1-(4-Trifluoromethoxyphenyl)-3-[3-(6,7-dimethoxyquinazolin-4-yloxy)phenyl]thiourea* (**10f**). Off white solid, yield: 54.5%, m.p. 171.5–174.0 °C, purity: 98.7% (HPLC). ^1^H-NMR (400 MHz, DMSO-*d*_6_) δ 3.97 (s, 6H, quinazoline -OCH_3_), 7.06 (dd, *J* = 2.1, 7.8 Hz, 1H), 7.32 (d, *J* = 8.6 Hz, 2H), 7.37 (s, 1H), 7.42 (t, *J* = 8.2 Hz, 1H), 7.53 (d, *J* = 10.8 Hz, 2H), 7.66–7.73 (m, 3H), 8.55 (s, 1H), 10.75 (s, 1H, -NHCS-), 10.81 (s, 1H, -NHCS-). HRMS (AP-ESI) *m*/*z*: calcd. for C_24_H_20_F_3_N_4_O_4_S [M + H]^+^ 517.1157, found 517.1151.

*1-(2,4-Dichlorophenyl)-3-[3-(6,7-dimethoxyquinazolin-4-yloxy)phenyl]thiourea* (**10g**). Off white solid, yield: 65.5%, m.p. 187.4–188.2 °C, purity: 98.3% (HPLC). ^1^H-NMR (400 MHz, DMSO-*d*_6_) δ 3.98 (s, 3H, quinazoline -OCH_3_-6), 3.99 (s, 3H, quinazoline -OCH_3_-7), 7.09-7.13 (m, 1H), 7.40 (s, 1H), 7.41–7.48 (m, 3H), 7.56 (s, 1H), 7.59 (s, 1H), 7.62 (s, 1H), 7.69 (d, *J* = 2.3 Hz, 1H), 8.56 (s, 1H), 9.60 (s, 1H, -NHCS-), 10.19 (s, 1H, -NHCS-). HRMS(AP-ESI) *m*/*z*: calcd. for C_23_H_19_Cl_2_N_4_O_3_S [M + H]^+^ 501.0555, found 501.0550.

*1-(3,4-Difluorophenyl)-3-[3-(6,7-dimethoxyquinazolin-4-yloxy)phenyl]thiourea* (**10h**). Light grey solid, yield: 47.6%, m.p. 181.3–184.1 °C, purity: 98.0% (HPLC). ^1^H-NMR (400 MHz, DMSO-*d*_6_) δ 3.96 (s, 3H, quinazoline -OCH_3_-6), 3.97 (s, 3H, quinazoline -OCH_3_-7), 7.08 (dd, *J* = 2.5, 8.4 Hz, 1H), 7.22 (ddt, *J* = 2.2, 4.3, 8.6 Hz, 1H), 7.33–7.47 (m, 4H), 7.50–7.60 (m, 2H), 7.69 (dd, *J* = 2.5, 8.6 Hz, 1H), 8.55 (s, 1H), 10.06 (s, 1H, -NHCS-), 10.12 (s, 1H, -NHCS-). HRMS(AP-ESI) *m*/*z*: calcd. for C_23_H_19_F_2_N_4_O_3_S [M + H]^+^ 469.1146, found 469.1149.

*1-(4-Chlorophenyl)-3-[3-(6,7-dimethoxyquinazolin-4-yloxy)phenyl]thiourea* (**10i**). Off white solid, yield: 74.7%, m.p. 198.3–201.0 °C, purity: 98.1% (HPLC). ^1^H-NMR (400 MHz, DMSO-*d*_6_) δ 3.97 (s, 3H, quinazoline -OCH_3_-6), 3.98 (s, 3H, quinazoline -OCH_3_-7), 7.05 (dd, *J* = 2.5, 8.3 Hz, 1H), 7.34–7.45 (m, 4H), 7.46–7.51 (m, 1H), 7.53–7.61 (m, 3H), 7.64 (d, *J* = 2.5 Hz, 1H), 8.55 (s, 1H), 10.55 (s, 1H, -NHCS-), 10.62 (s, 1H, -NHCS-). HRMS(AP-ESI) *m*/*z*: calcd. for C_23_H_20_ClN_4_O_3_S [M + H]^+^ 467.0945, found 467.0948

*1-(4-Fluorophenyl)-3-[3-(6,7-dimethoxyquinazolin-4-yloxy)phenyl]thiourea* (**10j**). Off white solid, yield: 53.0%, m.p. 168.5–170.0 °C, purity: 98.3% (HPLC). ^1^H-NMR (400 MHz, DMSO-*d*_6_) δ 3.96 (s, 3H, quinazoline -OCH_3_-6), 3.98 (s, 3H, quinazoline -OCH_3_-7), 7.05 (dd, *J* = 2.0, 8.1 Hz, 1H), 7.12–7.20 (m, 2H), 7.38 (s, 1H), 7.39–7.46 (m, 2H), 7.48–7.49 (m, 2H), 7.54 (s, 1H), 7.62 (d, *J* = 2.4 Hz, 1H), 8.55 (s, 1H), 10.21 (s, 1H, -NHCS-), 10.32 (s, 1H, -NHCS-). HRMS(AP-ESI) *m*/*z*: calcd. for C_23_H_20_FN_4_O_3_S [M + H]^+^ 451.1240, found 451.1244.

*1-p-tolyl-3-[3-(6,7-dimethoxyquinazolin-4-yloxy)phenyl]thiourea* (**10k**). Off white solid, yield: 40.1%, m.p. 138.4–140.0 °C, purity: 99.2% (HPLC). ^1^H-NMR (400 MHz, DMSO-*d*_6_) δ 2.28 (s, 3H, Ph’-CH_3_), 3.99 (s, 6H, -OCH_3_), 7.04–7.08 (m, 1H), 7.14 (d, *J* = 8.2Hz, 2H), 7.33 (d, *J* = 8.2 Hz, 2H), 7.38 (s, 1H), 7.41 (s, 1H), 7.43 (s, 1H), 7.55 (s, 1H), 7.59 (s, 1H), 8.56 (s, 1H), 9.80 (s, 1H, -NHCS-), 9.81 (s, 1H, -NHCS-). HRMS(AP-ESI) *m*/*z*: calcd. for C_24_H_23_N_4_O_3_S [M + H]^+^ 447.1491, found 447.1497.

*1-[3-(6,7-Dimethoxyquinazolin-4-ylsulfanyl)phenyl]-3-(4-fluoro-3-trifluoromethylphenyl) thiourea* (**10l**). White solid, yield: 70.1%, m.p. 128.4–130.0 °C, purity: 98.2% (HPLC). ^1^H-NMR (400 MHz, DMSO-*d*_6_) δ 3.99 (s, 6H, -OCH_3_), 7.33 (s, 1H), 7.35 (s, 1H), 7.42 (d, *J* = 7.8Hz, 1H), 7.46–7.52 (m, 2H), 7.55 (d, *J* = 7.8Hz, 1H), 7.76–7.80 (m, 2H), 7.92 (dd, *J* = 2.6, 6.4Hz, 1H), 8.68 (s, 1H), 10.04 (s, 1H, -NHCS-), 10.16 (s, 1H, -NHCS-). HRMS(AP-ESI) *m*/*z*: calcd. for C_24_H_19_F_4_N_4_O_2_S_2_ [M + H]^+^ 535.0886, found 535.0882.

*1-(4-Chloro-3-trifluoromethylphenyl)-3-[3-(6,7-dimethoxyquinazolin-4-ylsulfanyl)phenyl] thiourea* (**10m**). Off white solid, yield: 54.1%, m.p. 162.2–164.0 °C, purity: 98.8% (HPLC). ^1^H-NMR (400 MHz, DMSO-*d*_6_) δ 3.97 (s, 6H, -OCH_3_), 7.32 (d, *J* = 8.8 Hz, 2H), 7.41 (dd, *J* = 1.4, 7.8 Hz, 1H), 7.49 (t, *J* = 7.9 Hz, 1H), 7.60–7.70 (m, 2H), 7.74–7.83 (m, 2H), 8.07 (d, *J* = 2.6 Hz, 1H), 8.67 (s, 1H), 10.24 (s, 1H, -NHCS-), 10.29 (s, 1H, -NHCS-). HRMS(AP-ESI) *m*/*z*: calcd. for C_24_H_19_ClF_3_N_4_O_2_S_2_ [M + H]^+^ 552.0590, found 552.0593.

*1-(4-Trifluoromethoxyphenyl)-3-[3-(6,7-dimethoxyquinazolin-4-ylsulfanyl)phenyl]thiourea* (**10n**). Off white solid, yield: 47.2%, m.p. 138.0–139.3 °C, purity: 98.3% (HPLC). ^1^H-NMR (400 MHz, DMSO-*d*_6_) δ 3.97 (s, 6H, -OCH_3_), 7.29–7.34 (m, 4H), 7.36 (dd, *J* = 1.4, 7.8 Hz, 1H), 7.46 (d, *J* = 7.8 Hz, 1H), 7.69–7.76 (m, 2H), 7.82 (dd, *J* = 2.2, 8.4 Hz, 1H), 7.96 (s, 1H), 8.68 (s, 1H), 11.09 (s, 1H, -NHCS-), 11.15 (s, 1H, -NHCS-). HRMS(AP-ESI) *m*/*z*: calcd. for C_24_H_20_F_3_N_4_O_3_S_2_ [M + H]^+^ 533.0929, found 533.0931.

*1-(3,5-Ditrifluoromethylphenyl)-3-[3-(6,7-dimethoxyquinazolin-4-ylsulfanyl)phenyl]thiourea* (**10o**). Light yellow solid, yield: 43.8%, m.p. 168.2–171.0 °C, purity: 98.5% (HPLC). ^1^H-NMR (400 MHz, DMSO-*d*_6_) δ 3.98 (s, 6H, -OCH_3_), 7.32 (s, 1H), 7.34 (s, 1H), 7.45 (d, *J* = 7.8 Hz, 1H), 7.52 (t, *J* = 7.8 Hz, 1H), 7.66 (d, *J* = 7.8 Hz, 1H), 7.79–7.81 (m, 2H), 8.26 (s, 2H), 8.69 (s, 1H), 10.41 (s, 1H, -NHCS-), 10.46 (s, 1H, -NHCS-). HRMS(AP-ESI) *m*/*z*: calcd. for C_25_H_19_F_6_N_4_O_2_S_2_ [M + H]^+^ 585.0854, found 585.0857.

*1-(3-Trifluoromethylphenyl)-3-[3-(6,7-dimethoxyquinazolin-4-ylsulfanyl)phenyl]thiourea* (**10p**). Off white solid, yield: 49.4%, m.p. 127.5–131.0 °C, purity: 98.1% (HPLC). ^1^H-NMR (400 MHz, DMSO-*d*_6_) δ 3.98 (s, 6H, -OCH_3_), 7.32 (s, 1H), 7.34 (s, 1H), 7.41 (d, *J* = 7.8 Hz, 1H), 7.46–7.51 (m, 2H), 7.56 (t, *J* = 7.9 Hz, 1H), 7.66 (d, *J* = 7.8 Hz, 1H), 7.76 (d, *J* = 7.9 Hz, 1H), 7.80 (t, *J* = 1.7 Hz, 1H), 7.95 (s, 1H), 8.69 (s, 1H), 10.17 (s, 1H, -NHCS-), 10.21 (s, 1H, -NHCS-). HRMS(AP-ESI) *m*/*z*: calcd. for C_24_H_20_F_3_N_4_O_2_S_2_ [M + H]^+^ 517.0980, found 517.0983.

*1-(4-Bromo-3-trifluoromethylphenyl)-3-[3-(6,7-dimethoxyquinazolin-4-ylsulfanyl)phenyl] thiourea* (**10q**). Off white solid, yield: 52.3%, m.p. 150.3–152.0 °C, purity: 98.4% (HPLC). ^1^H-NMR (400 MHz, DMSO-*d*_6_) δ 3.98 (s, 6H, -OCH_3_), 7.33 (s, 1H), 7.35 (s, 1H), 7.42 (d, *J* = 7.8 Hz, 1H), 7.50 (t, *J* = 7.8 Hz, 1H), 7.64 (d, *J* = 7.8 Hz, 1H), 7.71 (dd, *J* = 2.2, 8.8 Hz, 1H), 7.77 (s, 1H), 7.83 (d, *J* = 8.8 Hz, 1H), 8.07 (d, *J* = 2.2 Hz, 1H), 8.69 (s, 1H), 10.19 (s, 1H, -NHCS-), 10.26 (s, 1H, -NHCS-). HRMS(AP-ESI) *m*/*z*: calcd. for C_24_H_19_BrF_3_N_4_O_2_S_2_ [M + H]^+^ 596.0085, found 596.0080.

*1-(2,4-Dichlorophenyl)-3-[3-(6,7-dimethoxyquinazolin-4-ylsulfanyl)phenyl]thiourea* (**10r**). White solid, yield: 48.1%, m.p. 172.4–174.0 °C, purity: 98.5% (HPLC). 1H-NMR (400 MHz, DMSO-*d*_6_) δ 3.99 (s, 6H, -OCH_3_), 7.33 (s, 1H), 7.35 (s, 1H), 7.41 (d, *J* = 7.8 Hz, 1H), 7.43 (dd, *J* = 2.4, 8.6 Hz, 1H), 7.49 (s, 1H), 7.60 (d, *J* = 8.6 Hz, 1H), 7.68 (d, *J* = 2.4 Hz, 1H), 7.70 (d, *J* = 7.8 Hz, 1H), 7.85 (t, *J* = 1.7 Hz, 1H), 8.68 (s, 1H), 9.60 (s, 1H, -NHCS-), 10.19 (s, 1H, -NHCS-). HRMS(AP-ESI) *m*/*z*: calcd. for C_23_H_19_Cl_2_N_4_O_2_S_2_ [M + H]^+^ 518.0326, found 518.0322.

*1-(3,4-Difluorophenyl)-3-[3-(6,7-dimethoxyquinazolin-4-ylsulfanyl)phenyl]thiourea* (**10s**). Off white solid, yield: 45.4%, m.p. 128.2–130.4 °C, purity: 98.0% (HPLC). ^1^H-NMR (400 MHz, DMSO-*d*_6_) δ 3.97 (s, 6H, -OCH_3_), 7.31 (t, *J* = 4.7 Hz 3H), 7.35–7.42 (m, 2H), 7.46 (d, *J* = 7.9 Hz, 1H), 7.80 (dd, *J* = 2.1, 7.8 Hz, 1H), 7.97–7.88 (m, 2H), 8.68 (s, 1H), 10.11 (s, 2H, -NHCS-). HRMS (AP-ESI) *m*/*z*: calcd. for C_23_H_19_F_2_N_4_O_2_S_2_ [M + H]^+^ 485.0917, found 485.0918.

*1-(4-Chlorophenyl)-3-[3-(6,7-dimethoxyquinazolin-4-ylsulfanyl)phenyl]thiourea* (**10t**). Off white solid, yield: 47.1%, m.p. 124.8–126.1 °C, purity: 98.1% (HPLC). ^1^H-NMR (400 MHz, DMSO-*d*_6_) δ 3.98 (s, 6H, -OCH_3_), 7.30 (d, *J* = 8.6 Hz, 2H), 7.35–7.38 (m, 3H), 7.43–7.53 (m, 3H), 7.65 (dd, *J* = 2.3, 8.4 Hz, 1H), 7.79 (t, *J* = 2.4 Hz, 1H), 8.67 (s, 1H), 10.01 (s, 1H, -NHCS-), 10.04 (s, 1H, -NHCS-). HRMS(AP-ESI) *m*/*z*: calcd. for C_23_H_20_ClN_4_O_2_S_2_ [M + H]^+^ 484.0716, found 484.0712.

*1-(4-Fluorophenyl)-3-[3-(6,7-dimethoxyquinazolin-4-ylsulfanyl)phenyl]thiourea* (**10u**). Off white solid, yield: 40.6%, m.p. 152.0–153.2 °C, purity: 98.0% (HPLC). ^1^H-NMR (400 MHz, DMSO-*d*_6_) δ 3.97 (s, 6H, -OCH_3_), 7.12–7.21 (m, 2H), 7.31 (d, *J* = 8.8 Hz, 2H), 7.34–7.40 (m, 1H), 7.41–7.51 (m, 3H), 7.62–7.69 (m, 1H), 7.78 (t, *J* = 1.8 Hz, 1H), 8.67 (s, 1H), 9.88 (s, 1H, -NHCS-), 9.95 (s, 1H, -NHCS-). HRMS(AP-ESI) *m*/*z*: calcd. for C_23_H_20_FN_4_O_2_S_2_ [M + H]^+^ 467.1012, found 467.1015

*1-p-tolyl-3-[3-(6,7-dimethoxyquinazolin-4-ylsulfanyl)phenyl]thiourea* (**10v**). White solid, yield: 43.9%, m.p. 163.1–165.0 °C, purity: 98.2% (HPLC). ^1^H-NMR (400 MHz, DMSO-*d*_6_) δ 2.26 (s, 3H, Ph-CH_3_), 3.97 (s, 6H, -OCH_3_), 7.13 (d, *J* = 8.2 Hz, 2H), 7.30 (d, *J* = 3.2 Hz, 2H), 7.32 (d, *J* = 3.1 Hz, 2H), 7.34–7.39 (m, 1H), 7.45 (t, *J* = 7.8 Hz, 1H), 7.66 (dt, *J* = 1.4, 8.2 Hz, 1H), 7.80 (t, *J* = 2.0 Hz, 1H), 8.67 (s, 1H), 9.84 (s, 1H, -NHCS-), 9.85 (s, 1H, -NHCS-). HRMS(AP-ESI) *m*/*z*: calcd. for C_24_H_23_N_4_O_2_S_2_ [M + H]^+^ 463.1262, found 463.1263.

1H-NMR of compound **10a**–**v** are shown in Supplementary Materials.

### 3.2. EGFR and VEGFR-2 Inhibitory Assay

Materials: EGFR and VEGFR-2 were purchased from Carna Biosciences (Framingham, MA, USA). ATP, DMSO and EDTA were purchased from Sigma (CA, USA). 96-well plate and 384-well plate were purchased from Corning.

In vitro enzymatic activities of all the synthesized compounds against VEGFR-2 and EGFR were evaluated by a caliper mobility shift assay using sorafenib as a positive control. Each of these compounds was dissolved in DMSO at 10 mM, diluted to 50× of the final desired highest inhibitor concentration in reaction by 100% DMSO, and 100 μL of each dilution was added to one well on 96-well storage plate, and diluted by transferring 10 μL to 90 μL of 100% DMSO in the next well. Besides, 100 μL of 100% DMSO was added to two empty wells for no compound control and no enzyme control in the same 96-well plate respectively. The plate was marked as source plate. 10 μL of each compound was transferred from the source plate to a new 96-well plate, which was marked as the intermediate plate. Next, 90 μL of 1× kinase base buffer was added to each well of the intermediate plate, and mixed by shaking vigorously for 10 min. A total of 5 μL of each well from the 96-well intermediate plate was transferred to a 384-well plate in duplicate.

Kinase reaction: kinase in 1× kinase base buffer, FAM-labeled peptide and ATP in the 1× kinase base buffer were added, then 10 μL of 2.5× enzyme solution was added to each well of the 384-well assay plate. After incubating at room temperature for 10 min, 10 μL of 2.5× peptide solution was added to each well of the 384-well assay plate. The reaction was incubated at 28 °C for a specified period of time. Finally, the kinase reactions were quenched with 25 μL of EDTA and the plates were washed. The dates were collected and converted with the caliper program.

### 3.3. In Vitro Antiproliferative Activity Assay

HCT116, MCF-7 and B16 cell lines were plated on 96-well plates at a density of 5000 per well and incubated overnight. The cells were treated with compounds and sorafenib at final concentrations ranging from 0.5 to 200 μM, while control cells were treated with equal volume DMSO. After 48 h, 0.5% MTT (Amresco, Solon, OH, USA) solution was added to each well, and further incubation for 4 h, then cells were centrifuged at 2500 rpm for 15 min and removed from the culture medium. And add 150 μL DMSO to dissolve the formazan. After mixing for 5 min, optical density was detected at 570 nm on a microplate reader (Thermo, Waltham, MA, USA).

### 3.4. In Vivo Antitumor Activity Assay

Male C57BL/6J mice were employed to establish the xenograft tumor model by subcutaneous injection (S.C.) of 6 × 10^6^ B16 cells. Twenty-four hours after inoculation, the animal were administrated with compounds **10b**, **10m**, **10q**, sorafenib or 0.5% CMC-Na orally successively for 14 days. In the end of treatment, the mice were sacrificed and the tumor were peeled off and weighted. The inhibition rate of tumor growth was calculated. All animal protocols were conformed to the Guidelines for the Care and Use of Laboratory Animals approved by the Animal Care and Use Committee of Yantai University.

### 3.5. Molecular Docking Study

Molecular docking was carried out using a Sybyl/Surflex dock based on the crystal structures of EGFR (PDB ID: 2ITY) and VEGFR-2 (PDB ID: 4ASD), which were downloaded from the RCSB Protein Data Bank and prepared using Docking Suite. Hydrogen was added and minimized using the Tripos force field and Pullman charges, and the default setting were used. Compound **10q** was depicted with the Sybyl/Skeetch module (Tripos Inc.) and docked into the defined binging site without constraint. Molecular docking results were submitted and generated with Pymol.

## 4. Conclusions

In summary, a series of novel quinazoline- and thiourea-containing sorafenib analogs (**10a**–**v**) were designed and synthesized as EGFR and VEGFR-2 dual TK inhibitors. Most of the compounds exhibited good enzymatic activities and potent antiproliferative activities against HCT-116, MCF-7 and B16 cell lines, which suggested that the introduction of electron-withdrawing groups on terminal phenyl ring was more favorable for enzyme inhibitory activities and antiproliferative activities. Compounds **10b** and **10q**, which exhibited the most potent inhibitory activities against EGFR (IC_50_ = 0.02 µM and 0.01 µM, respectively), VEGFR-2 (IC_50_ = 0.05 µM and 0.08 µM, respectively), and good antiproliferative activities, also displayed better anti-tumor activities than sorafenib in vivo by B16 melanoma xenograft model test. Furthermore, molecular docking of the most potent inhibitor **10q** into the ATP-binding site of EGFR and VEGFR-2 was performed and the result suggested that compound **10q** could bind well with the active site of EGFR and VEGFR-2. Therefore, compounds **10b** and **10q** could be developed as potent anticancer agents in the future.

## Figures and Tables

**Table 1:**
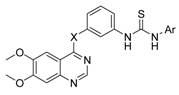
Enzymatic and cellular results of the target compounds.

Comp	Subsituent		IC_50_ (µM) ^a^		IC_50_ (µM) ^a^		
NO.	X	Ar	EGFR	VEGER-2	HCT116	MCF-7	B16
**10a**	O	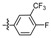	0.04	0.19	37.36	38.15	16.95
**10b**	O	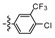	0.02	0.05	9.13	17.72	6.11
**10c**	O	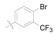	0.05	0.18	10.03	22.36	9.68
**10d**	O	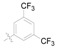	N.D.	N.D.	15.02	18.82	8.49
**10e**	O	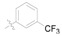	0.14	0.35	12.16	13.30	14.76
**10f**	O	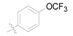	>10	>10	58.61	18.79	53.95
**10g**	O	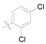	7.71	>10	19.83	17.09	24.47
**10h**	O	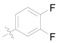	4.88	7.06	49.51	15.99	19.38
**10i**	O	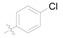	4.17	>10	>100	22.14	27.35
**10j**	O	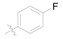	5.90	7.11	89.66	23.59	34.13
**10k**	O	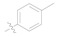	>10	>10	>100	26.48	>100
**10l**	S	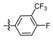	N.D.	N.D.	8.79	24.96	9.33
**10m**	S	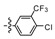	0.01	0.07	8.11	20.91	7.89
**10n**	S	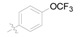	>10	>10	64.47	32.43	68.37
**10o**	S	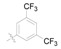	0.02	0.09	9.57	19.02	12.25
**10p**	S	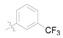	0.07	0.27	9.03	21.04	12.94
**10q**	S	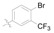	0.01	0.08	8.35	15.66	5.57
**10r**	S	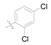	3.56	>10	63.10	14.79	27.39
**10s**	S	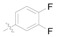	3.17	5.14	>100	30.53	42.16
**10t**	S	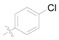	2.86	6.93	23.09	26.40	57.61
**10u**	S	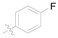	5.35	6.62	27.26	21.31	61.23
**10v**	S	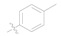	>10	>10	83.25	28.87	>100
sorafenib			0.02	0.08	10.55	17.87	9.29

N.D. not determined; ^a^ Assays were performed in triplicate (*n* ≥ 3).

**Table 2:** The effect of **10b**, **10m**, **10q** and sorafenib on the growth of B16 xenograft model.

Groups	Dosage (mg/kg)	Number Initial/End	Body Weight (g)		Tumor Weight (g)	Inhibition Rate (%)
			Initial	End		
Control	0	6/6	19.6 ± 2.3	21.8 ± 2.3	1.28 ± 0.56	
Sorafenib	90	6/6	20.7 ± 2.1	21.3 ± 2.4	0.88 ± 0.46	31.25%
**10b**	90	6/6	18.9 ± 1.8	19.5 ± 3.0	0.65 ± 0.22 *	49.22%
**10m**	90	6/6	19.2 ± 2.1	20.9 ± 1.6	1.02 ± 0.36 *	20.31%
**10q**	90	6/6	19.1 ± 2.7	20.2 ± 2.7	0.46 ± 0.14 *	64.06%

Antitumor effect is assessed by i.p. treatment of C57BL/6J mice carrying B16 tumor xenografts with 90 mg/kg of test compounds, sorafenib and CMC-Na as control. * *p* < 0.05, compared with sorafenib.

## Supplementary Materials

The supplementary materials are available online, 1H-NMR of compound **10a**–**v**.
