# Supplementary material for: Design and Discovery of Quinazoline- and Thiourea-Containing Sorafenib Analogs as EGFR and VEGFR-2 Dual TK Inhibitors
Source: Molecules. 2017 Dec 23;23(1):24. doi: 10.3390/molecules23010024 (PMC5943947; doi:10.3390/molecules23010024)

Compound 10a

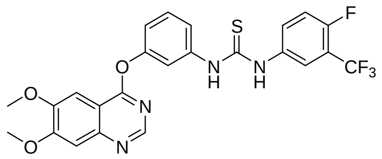

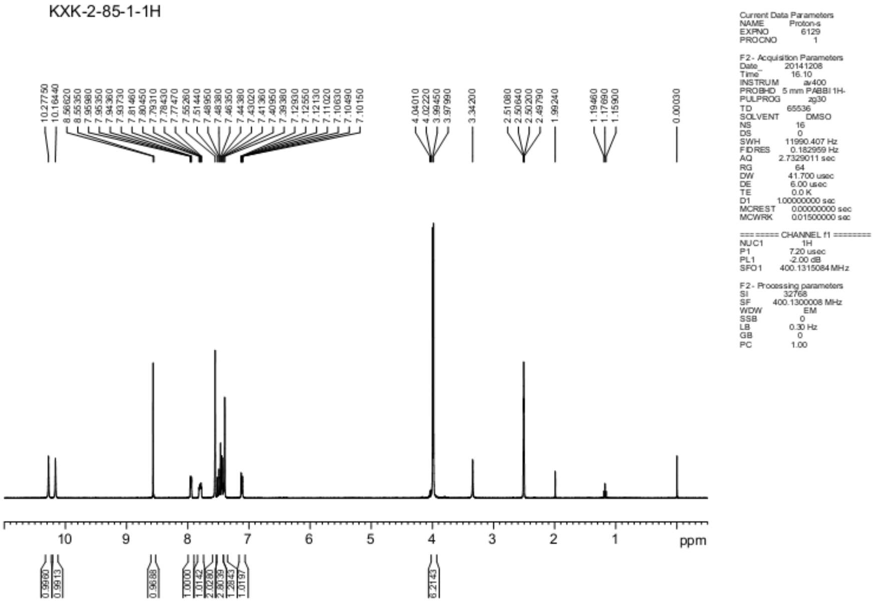

Compound 10b

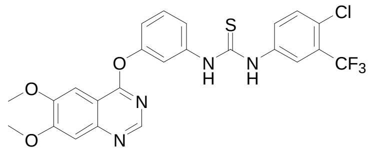

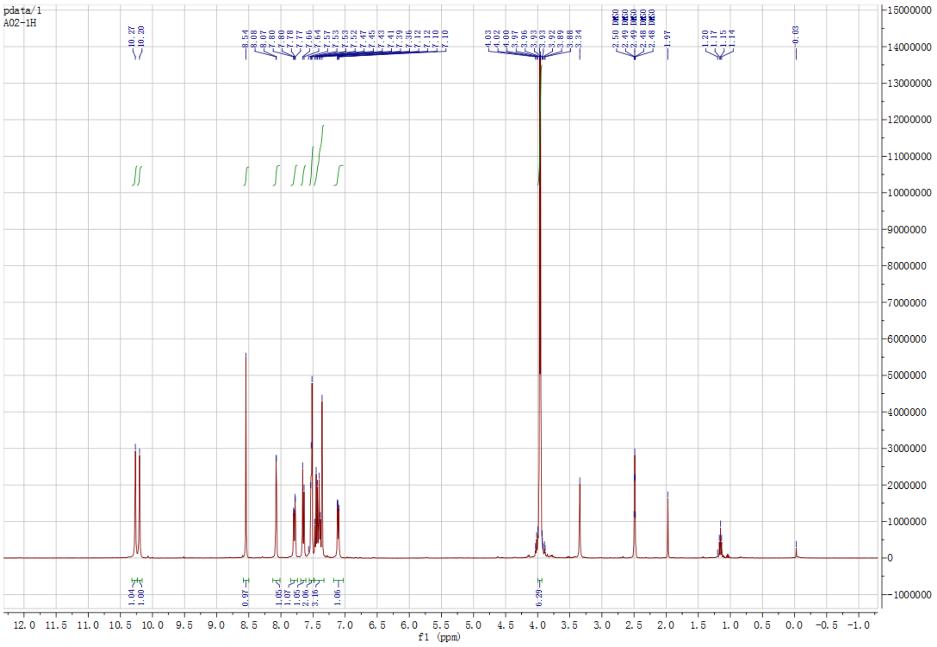

Compound 10c

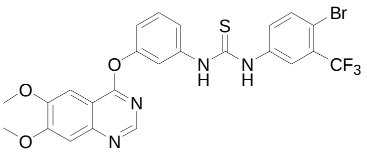

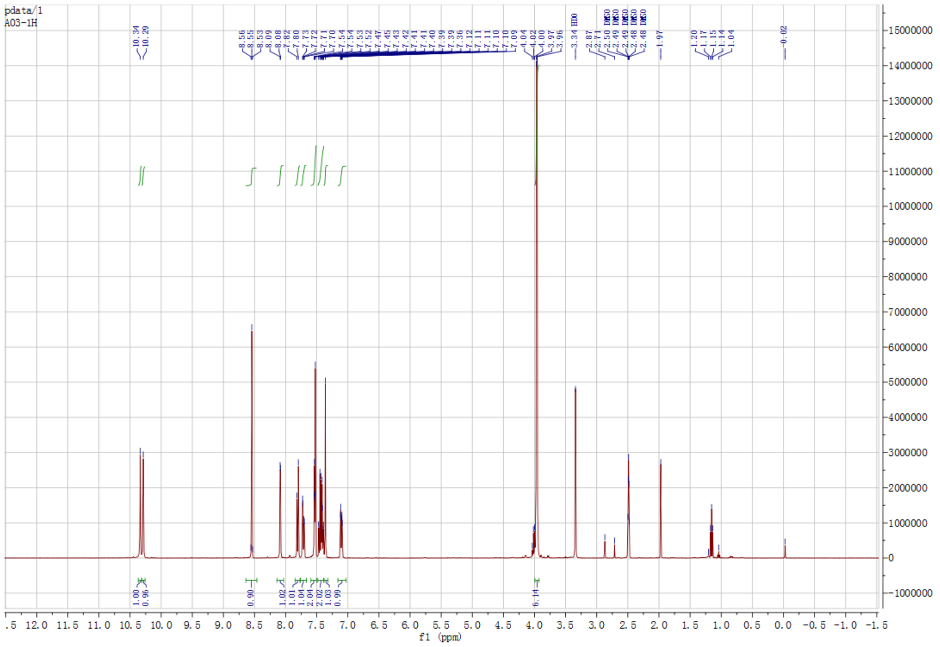

Compound 10d

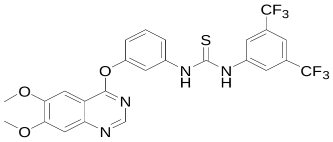

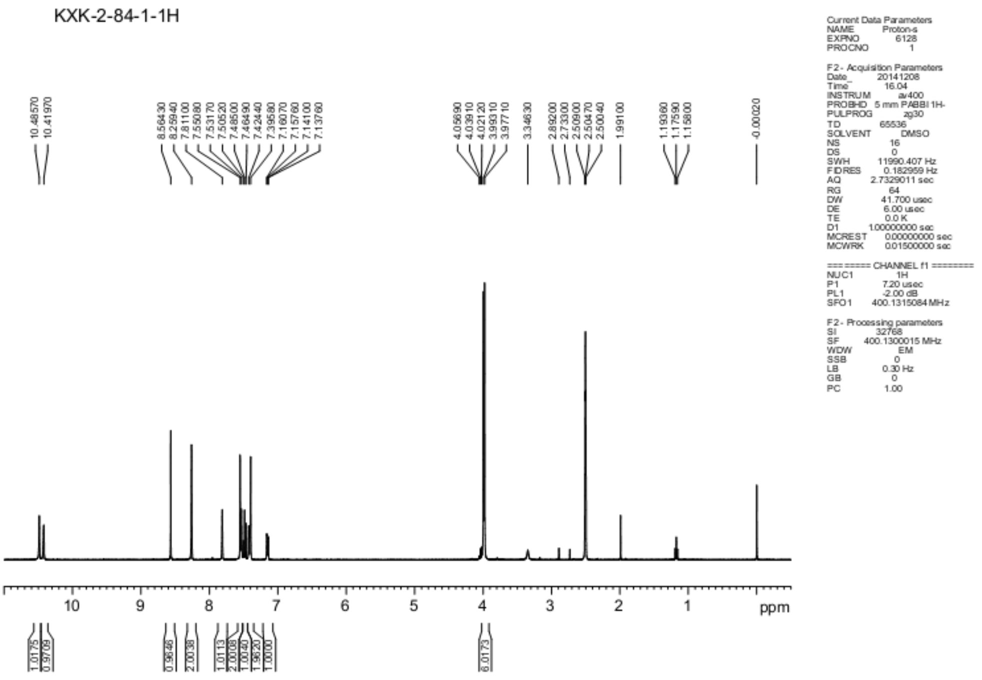

Compound 10e

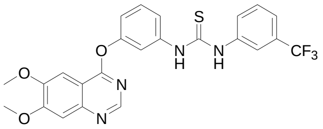

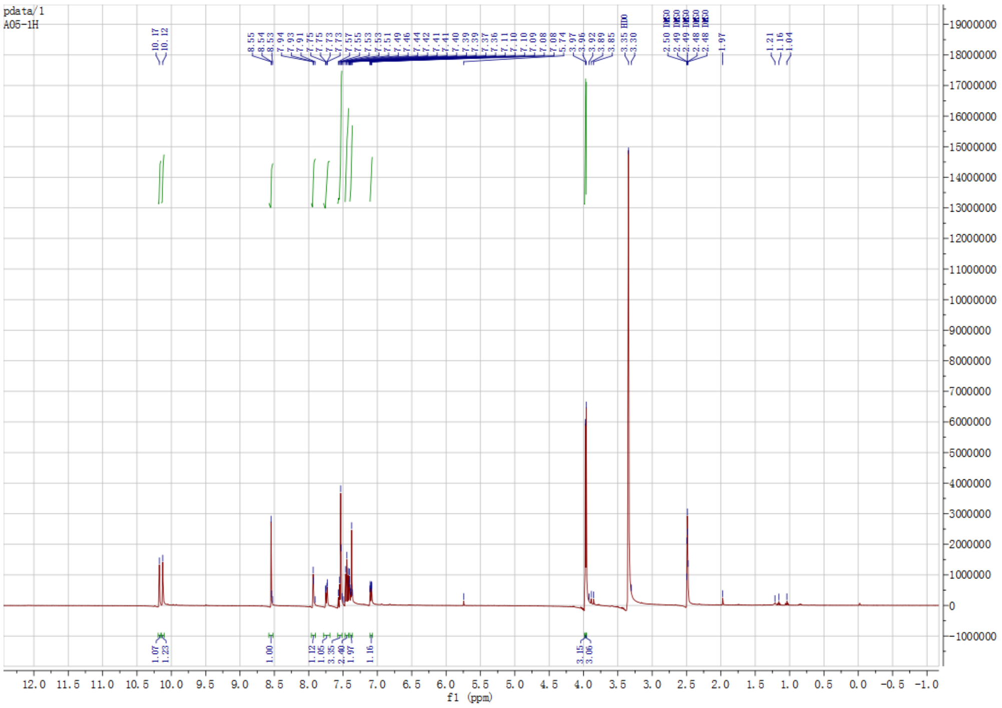

Compound 10f

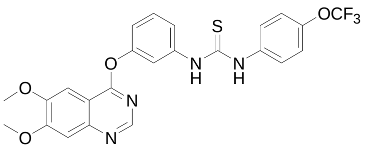

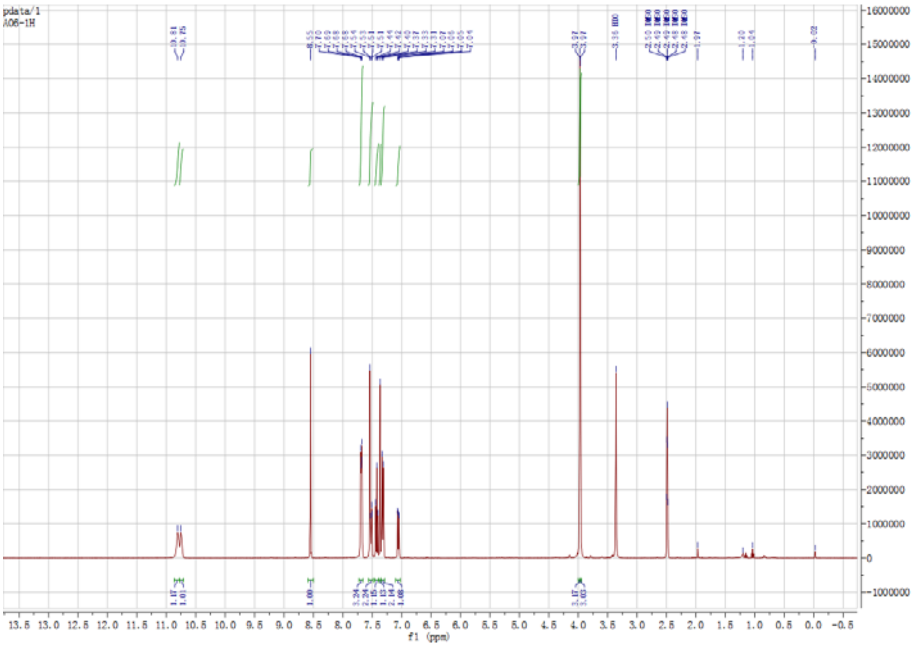

Compound 10g

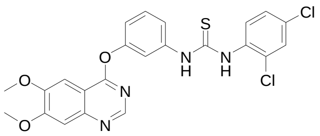

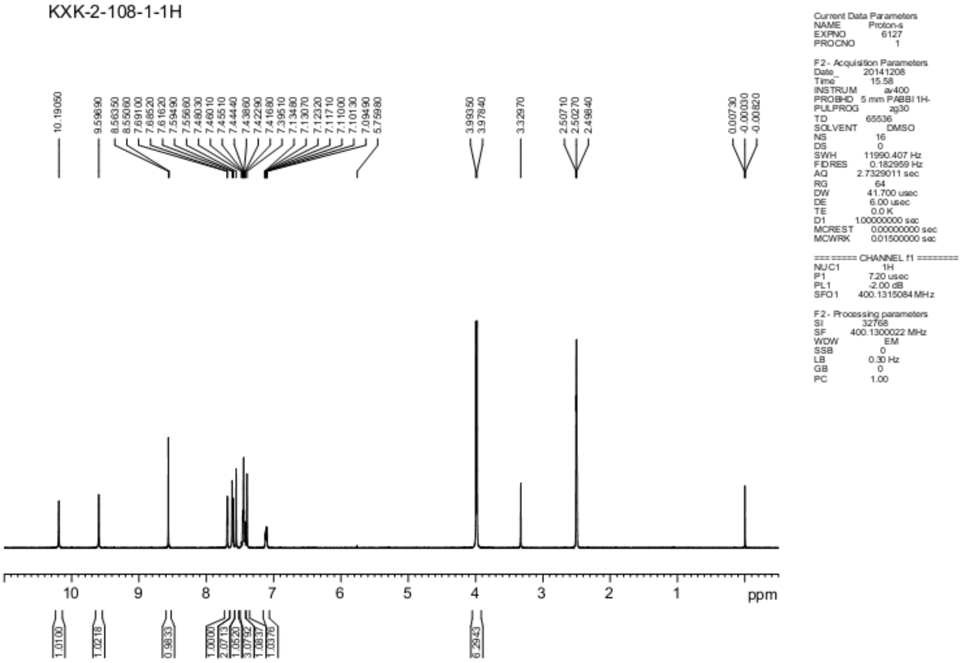

Compound 10h

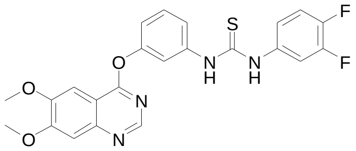

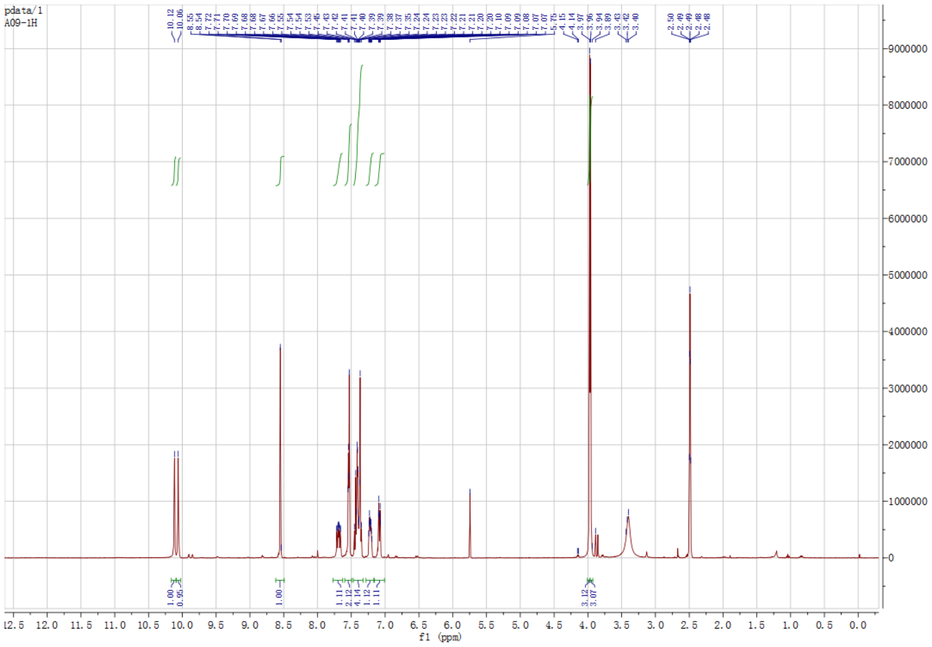

Compound 10i

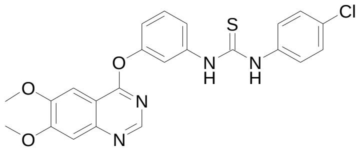

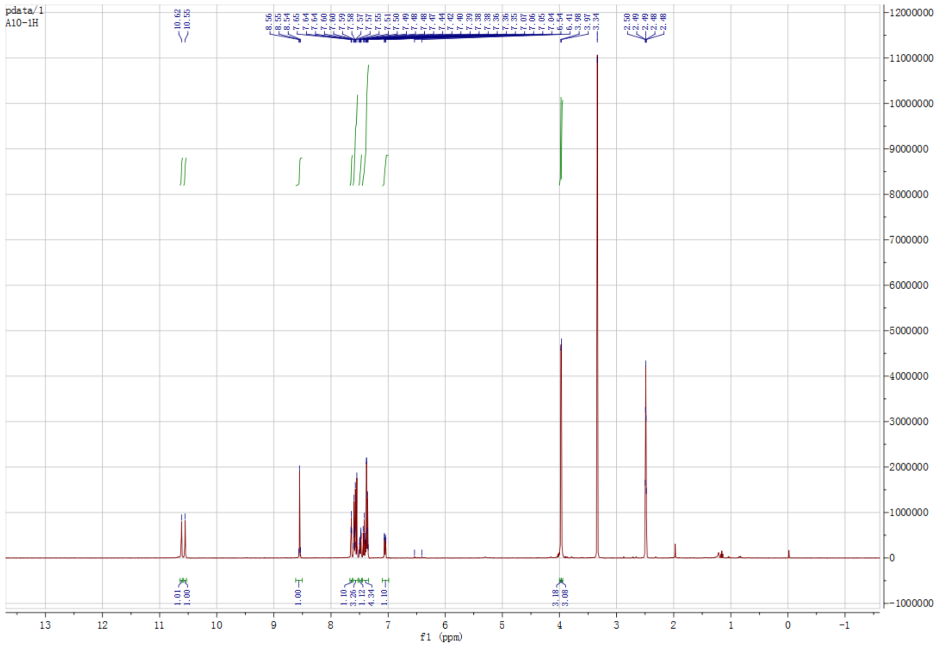

Compound 10j

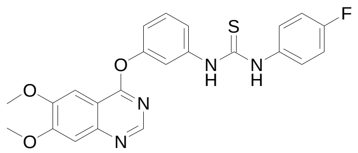

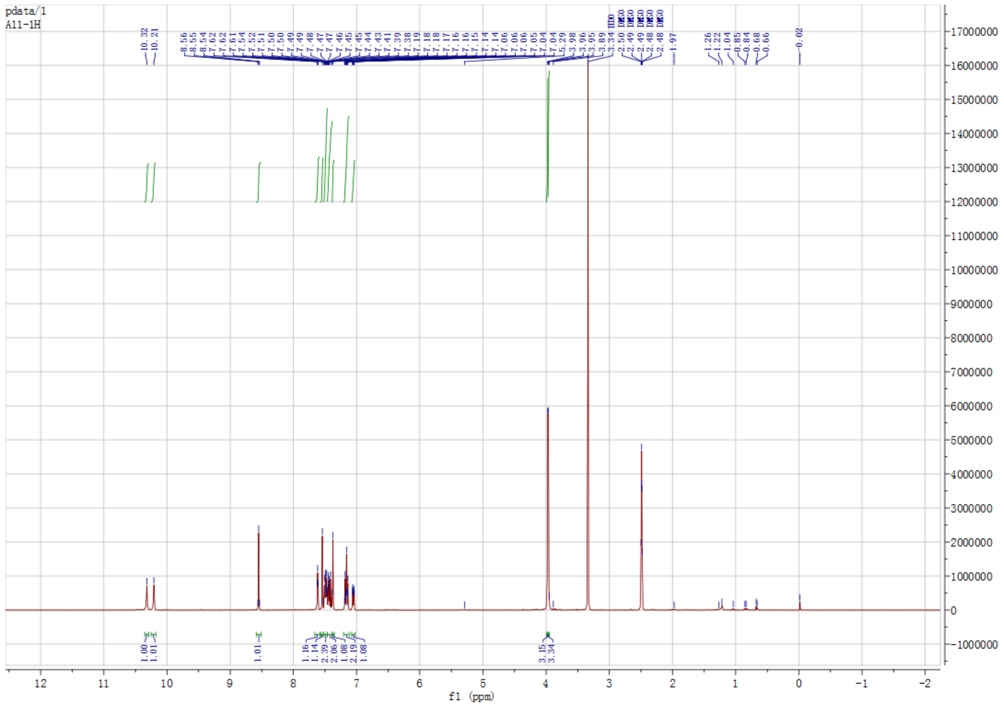

Compound 10k

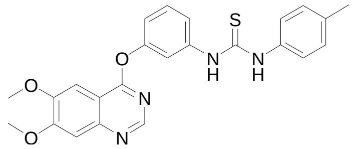

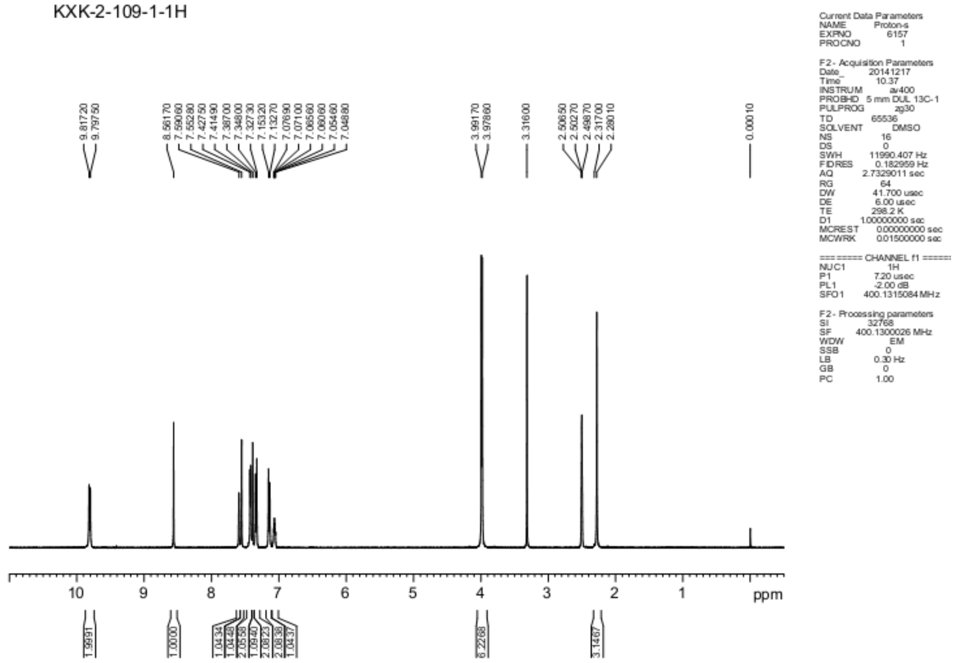

Compound 10l

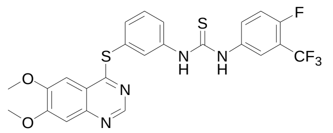

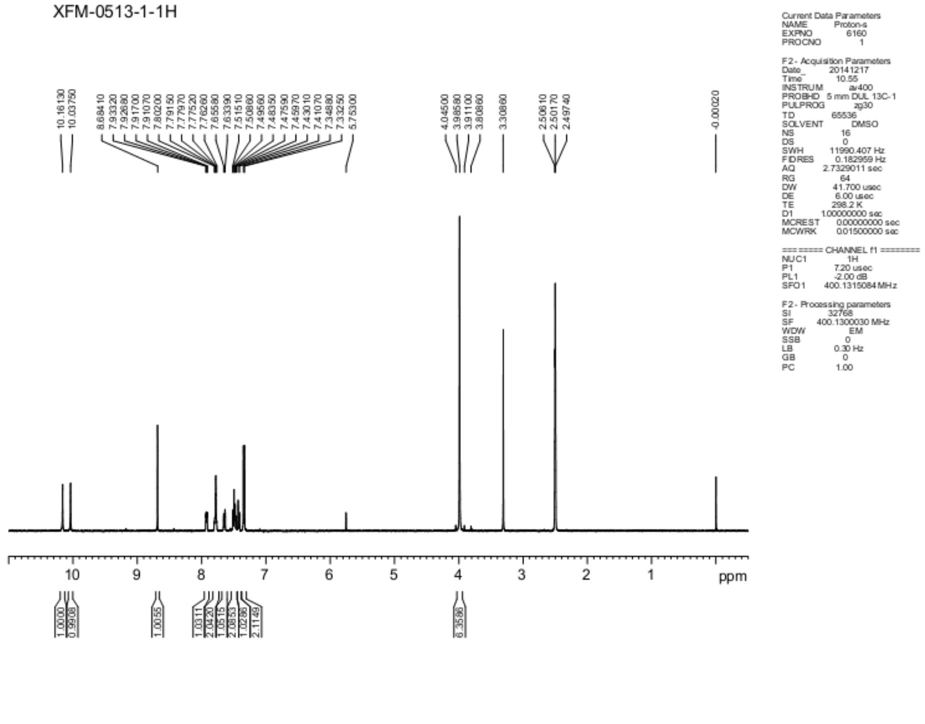

Compound 10m

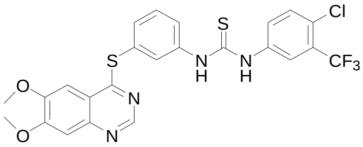

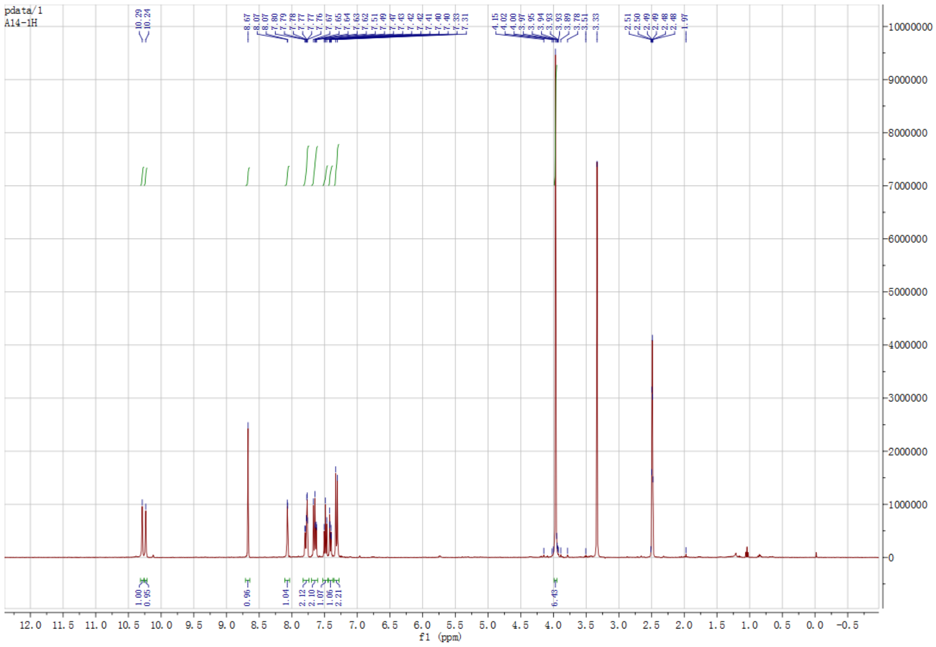

Compound 10n

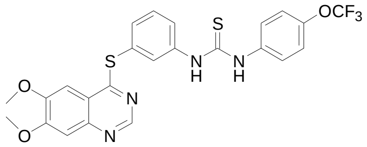

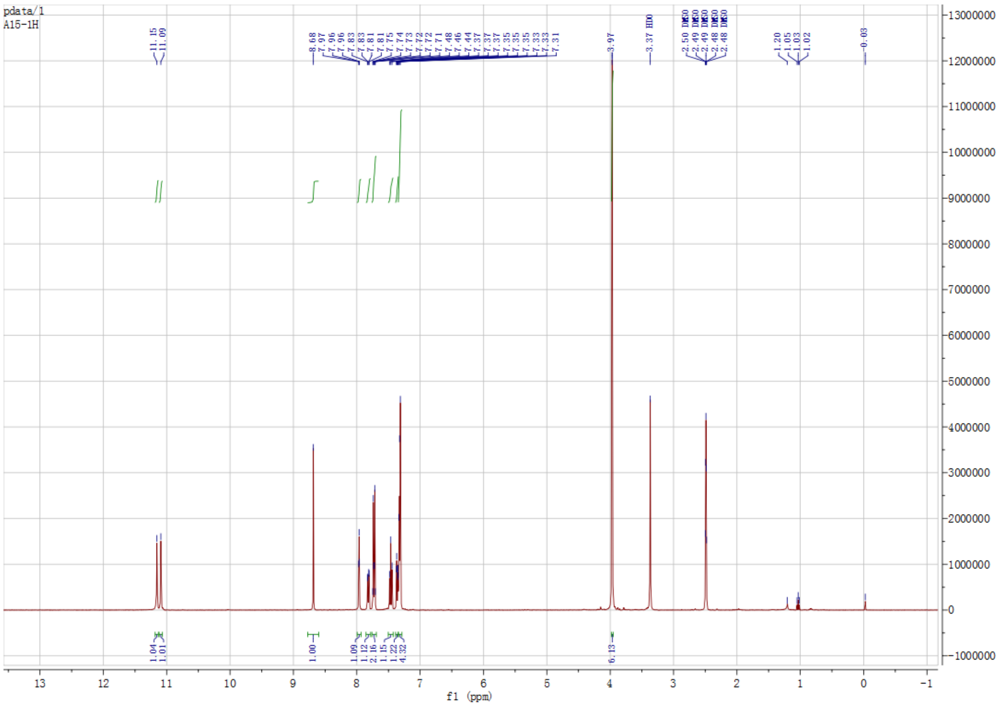

Compound 10o

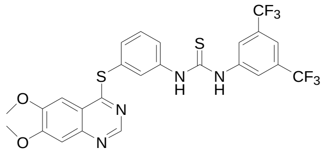

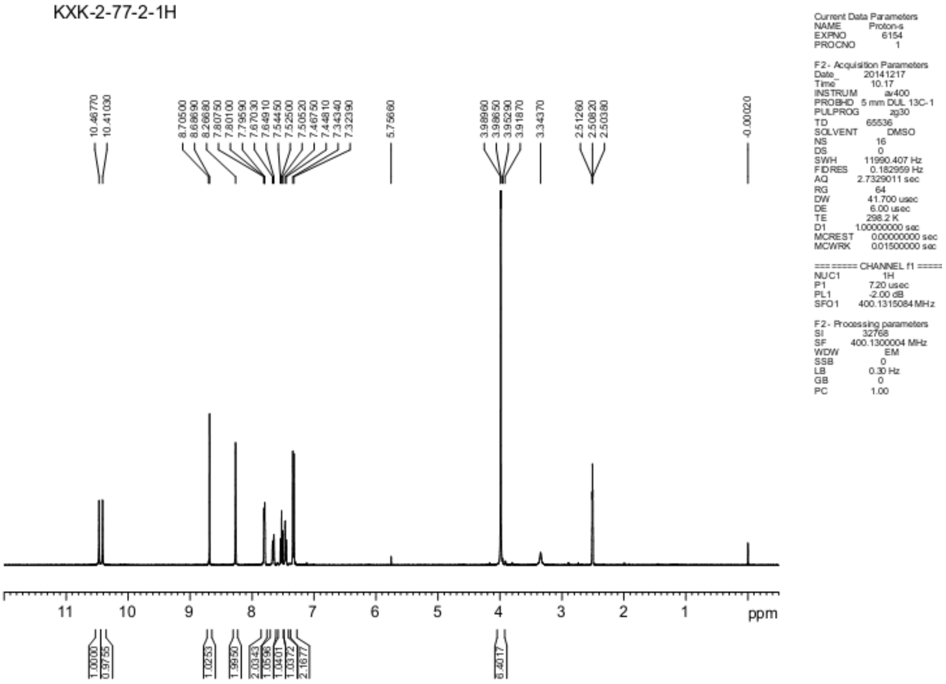

Compound 10p

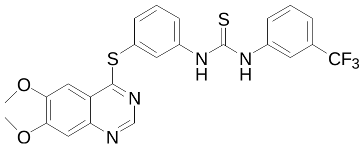

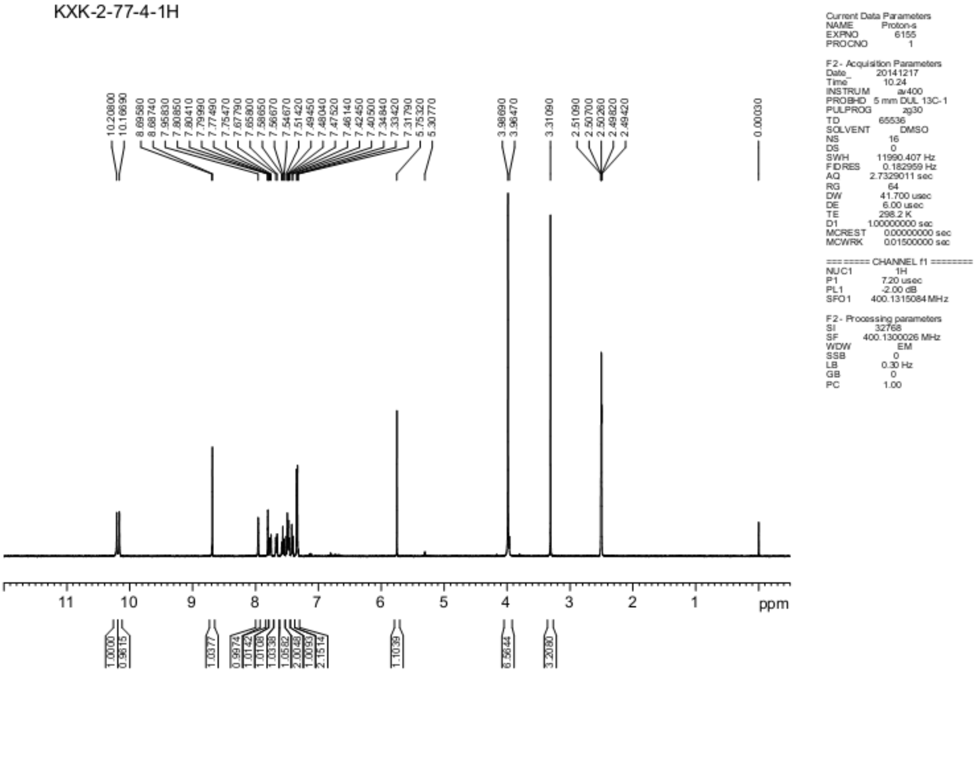

Compound 10q

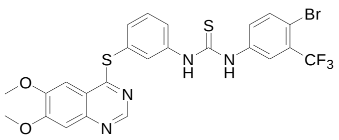

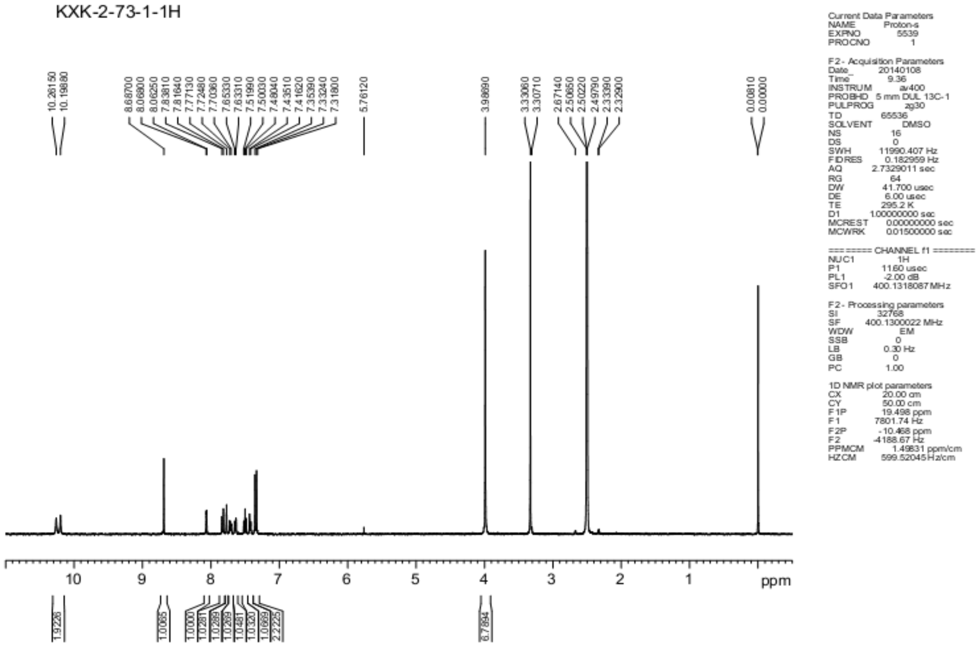

Compound 10r

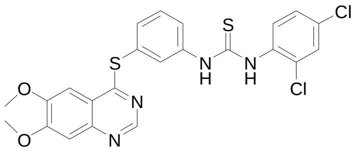

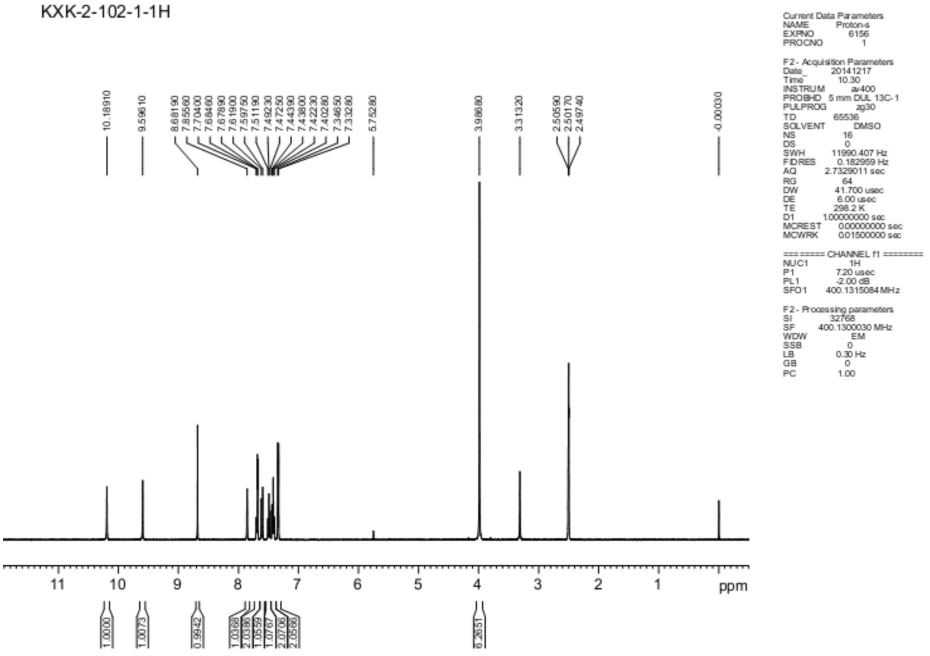

Compound 10s

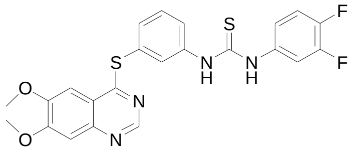

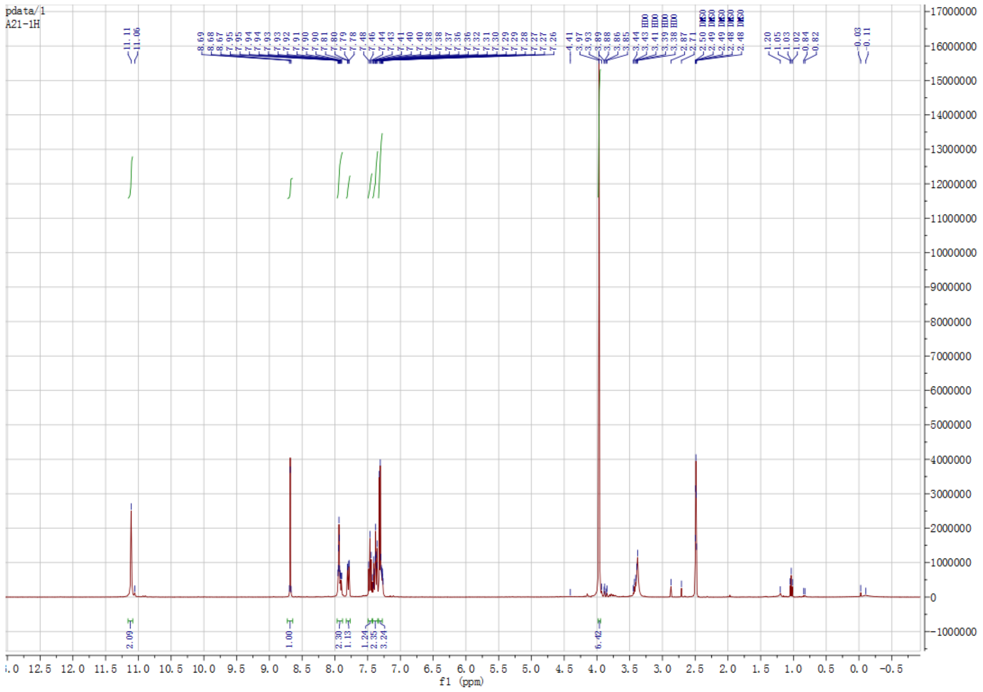

Compound 10t

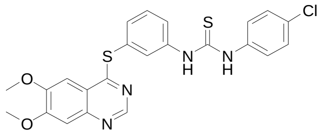

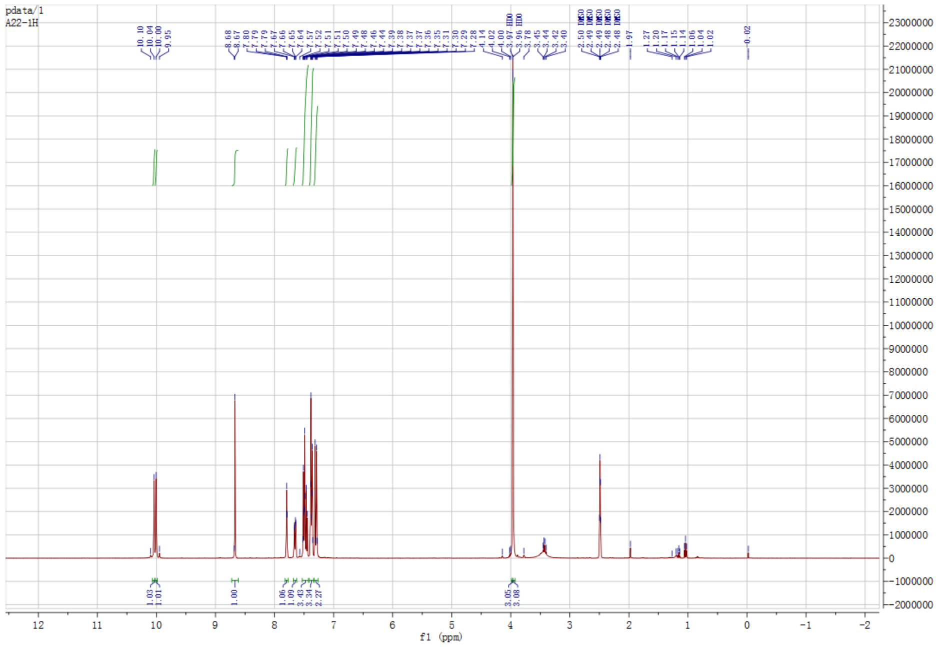

.

Compound 10u

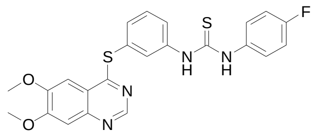

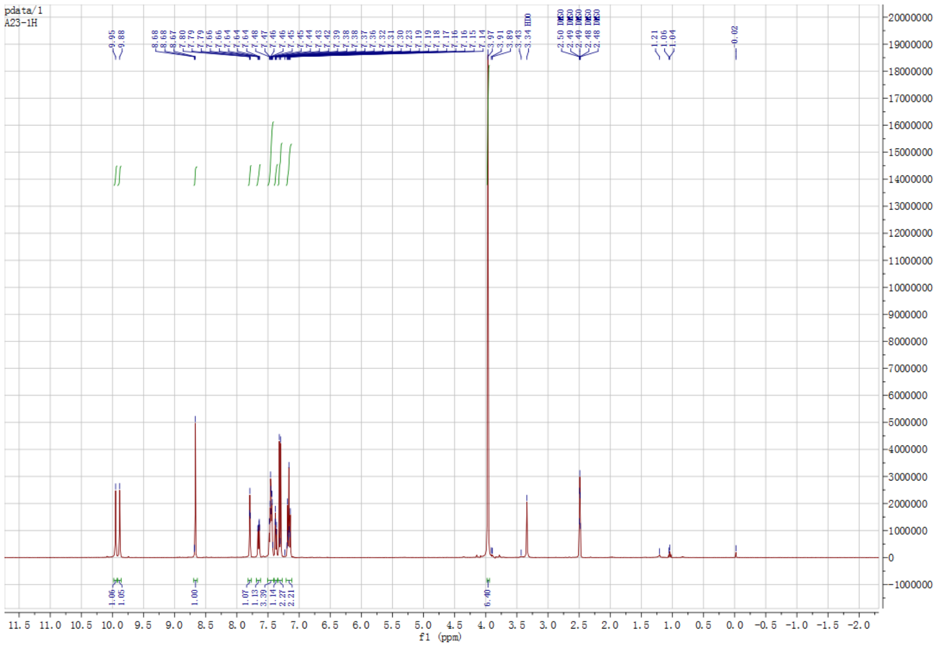

Compound 10v

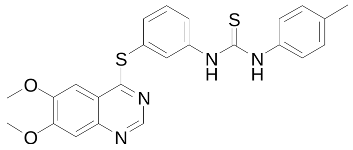

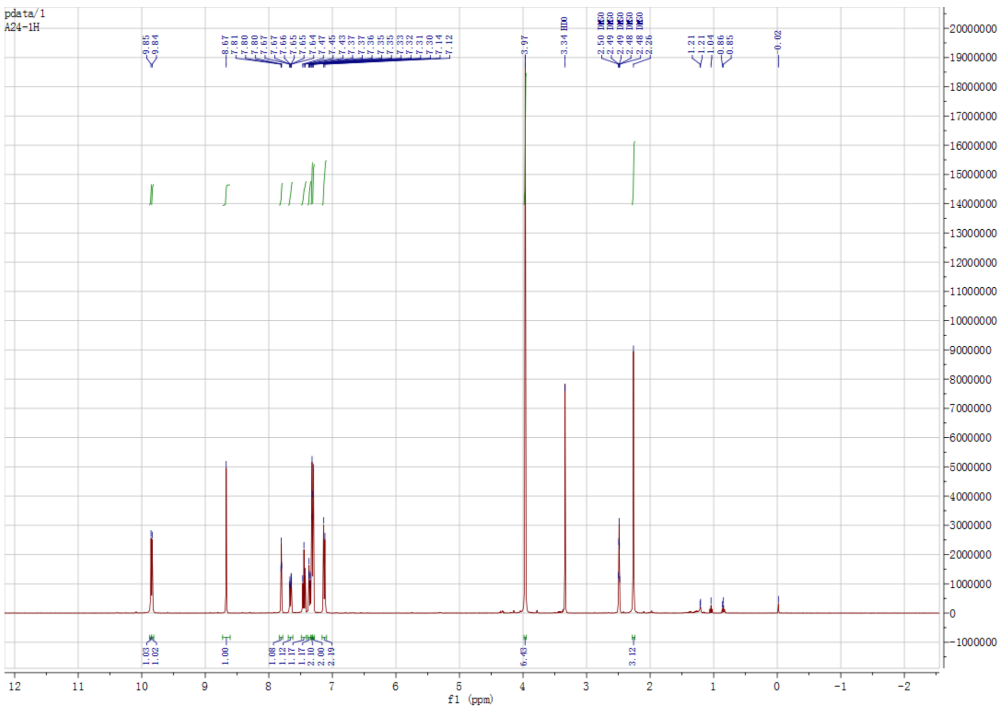

Supplement: Supplementary file 1 [file molecules-23-00024-s001.pdf]
